# Characteristics of Amino Acid and Glucose Digestion and Metabolism in Energy and Protein Feedstuffs for Pigs

**DOI:** 10.3390/ani15111510

**Published:** 2025-05-22

**Authors:** Jiayu Tu, Qingyun Chen, Junyan Zhou, Yuxin Fan, Yanlong Li, Yonghang Ma, Xiangfang Zeng, Shiyan Qiao, Shuang Cai

**Affiliations:** 1 State Key Laboratory of Animal Nutrition and Feeding, Ministry of Agriculture and Rural Affairs Feed Industry Centre, China Agricultural University, Beijing 100193, China; tujiayu0302@163.com (J.T.); chenqingyun1999@126.com (Q.C.); fyuxinfan@163.com (Y.F.); liyanlong1448@163.com (Y.L.); myh1602@foxmail.com (Y.M.); zengxf@cau.edu.cn (X.Z.); qiaoshiyan@cau.edu.cn (S.Q.); 2 Frontier Technology Research Institute of China Agricultural University in Shenzhen, Shenzhen 518116, China; 3 College of Animal Science and Technology, Beijing University of Agriculture, Beijing 102206, China; zhoujunyan@bua.edu.cn

**Keywords:** glucose, amino acid, feedstuff, digestion, pig

## Abstract

The objective of this study is to investigate the digestion and metabolism characteristics of amino acids and glucose in energy and protein feeds, and to establish regression equations to accurately predict their release rates in vivo based on the in vitro digestion characteristics of the feedstuffs. A total of 9 energy feedstuffs and 19 protein feedstuffs were selected for in vitro digestion simulation experiments. Additionally, four representative energy and protein feeds were then chosen for the portal vein, femoral artery, and femoral vein blood cannulation experiments in growing pigs. The results showed that among the nine energy feedstuffs tested, wheat bran presented the highest degree of digestion, followed by wheat, whereas potato had the lowest degree of digestion. The digestibility of starch at different time points in vitro was linearly correlated with the crude fiber content, total starch content, and ratio of amylose to amylopectin (R^2^ = 0.61~0.96). Among the 19 protein feedstuffs tested, peas released the highest total amount of amino acids, followed by sugar beet meal. The in vivo digestibility and metabolism trial in cannulated pigs showed that the total amino acid release was linearly correlated with the in vitro amino acid release rates, dry matter, crude protein, neutral detergent fiber, crude fat, and total energy of the feedstuffs (R^2^ = 0.93~0.99).

## Simple Summary

The release amounts and rates of glucose and amino acids in feedstuffs are crucial for the absorption and metabolism of nutrients in livestock and poultry. When the absorption of amino acids and energy is not synchronized, it leads to low nitrogen utilization efficiency in animals, resulting in resource wastage and environmental pollution. This study aimed to investigate the digestion and metabolism characteristics of amino acids and glucose in energy and protein feedstuffs. Among nine representative energy feedstuffs, wheat bran exhibited the highest degree of digestion, while potato had the lowest degree of digestion. The digestibility of starch in vitro was linearly correlated with the crude fiber content, total starch content, and the ratio of amylose to amylopectin. In the 19 protein feedstuffs tested, peas released the highest total amount of amino acids. The release of amino acids in vivo was linearly correlated with the in vitro amino acid release rates, dry matter, crude protein, neutral detergent fiber, crude fat, and gross energy of the feedstuffs. This study established a bridge between the in vitro digestion and in vivo metabolism of energy and protein feedstuffs, providing important data and tools for precise animal nutrition.

## 1. Introduction

Carbon and nitrogen are essential components of pig diets. However, the deposition efficiencies of carbon and nitrogen nutrients in pigs are only 32% and 52%, respectively [1]. This low efficiency results in significant resource waste and severe environmental pollution due to fecal waste [2,3]. These nutrients are primarily composed of energy and protein feedstuffs, and their sources and compositions determine their digestion and metabolic fate. Starch in feedstuffs undergoes physical digestion and enzymatic breakdown by amylase, maltase, and sucrase, ultimately yielding glucose for energy [4]. Proteins are hydrolyzed into free amino acids and oligopeptides by pepsin and trypsin, which are then absorbed by the small intestine and converted into proteins and hormones [5,6]. The different digestion rates of starch and protein alter the release kinetics of glucose and amino acids [7,8,9,10]. Therefore, how to select feedstuffs that synchronize the release of glucose and amino acids is crucial for improving nutrient absorption.

The release rates of glucose and amino acids are influenced by multiple factors, including feedstuff source, chemical composition, and structural characteristics. The composition, content, particle size, and ratio of amylose to amylopectin in starch affect the digestion, absorption, and metabolism of carbohydrates in animals [11,12]. Compared with amylose, amylopectin has a greater degree of gelatinization in the pig intestine, a larger surface area in contact with digestive enzymes, and faster digestion and absorption rates. Starches with lower amylose/amylopectin ratios are generally more easily digested and absorbed by livestock. Additionally, the interaction between starch and protein is a key factor affecting starch digestibility. The protein matrix encapsulates starch granules, limiting their swelling and reducing starch digestibility [13,14]. The factors influencing protein feedstuff digestibility include protein solubility, molecular structure, and processing methods. Different feedstuffs have varying protein solubilities and molecular compositions, resulting in different protein digestion rates [14,15]. In addition to properties of the protein itself, other dietary components such as cellulose and anti-nutritional factors also significantly influence protein digestion. Dietary fiber is mostly distributed in the cortex and unmilled grain kernels; the fibers are tightly wrapped around starches and proteins, forming a physical barrier that reduces their contact with digestive enzymes and decreases the digestibility [16]. And the presence of other anti-nutritional factors such as tannin, phytic acid, and saponin has been shown to decrease the protein digestibility [17,18]. The digestive properties of proteins may affect the metabolic fate of amino acids. Broilers fed with rapidly digestible protein had an increased average daily gain and feed conversion ratio than slowly digestible protein [19]. Maintaining the synchronized release of carbon and nitrogen in the intestine can effectively improve nitrogen utilization in feed. On the one hand, rapidly released glucose provides sufficient energy substrates for intestinal cells, reducing amino acid oxidation losses and increasing the amount of amino acids absorbed into the bloodstream [20,21,22]. On the other hand, the slowly digestible starch diet decreases enzyme activities in the small intestine and lowers the efficiency of protein synthesis [23]. However, systematic research on the release rates and kinetics of amino acids and glucose from feedstuffs in growing pigs is still lacking.

The objective of this study is to investigate the digestion and metabolism characteristics of amino acids and glucose in energy and protein feeds, and to establish regression equations to accurately predict their release rates in vivo based on the in vitro digestion characteristics of the feedstuffs.

## 2. Materials and Methods

### 2.1. Feedstuff Sources

A total of 9 different energy feedstuffs (corn, corn starch, sorghum, wheat, barley, extruded corn, potato, wheat bran, and cassava) and 19 different protein feedstuffs (soybean meal, extruded soybeans, peanut cake, distillers’ grains, fish meal, greens cake, whey protein powder, corn gluten meal, fermented soybean meal, palm kernel meal, soybean protein concentrate, rapeseed meal, sunflower meal, cottonseed meal, sprayed corn germ meal, hydrolyzed feather meal, beet meal, pea, and brewer’s yeast) were purchased from Wellhope Foods Co., Ltd. (Beijing, China) and Beijing Tonglixingke Agricultural Technology Co., Ltd. (Beijing, China) All the feedstuffs were milled to pass through a 40-mesh sieve.

### 2.2. Nutritional Composition Analysis of Feedstuffs

The nutritional compositions of various feedstuffs were comprehensively analyzed in duplicate. The gross energy (GE), dry matter (DM), crude protein (CP), ether extract (EE), crude fiber (CF), neutral detergent fiber (NDF), and acid detergent fiber (ADF) contents were determined for all the feedstuffs. In addition, for nine energy feedstuffs, total starch, amylose, and amylopectin contents were analyzed, and the ratio of amylopectin to amylose was calculated.

GE, DM, CP, EE, CF, NDF, and ADF were measured according to the Recommended National Standard of the People’s Republic of China. The total starch content was analyzed via the method recommended by the Association of Official Analytical Chemists. Amylose and amylopectin contents were determined via the dual-wavelength colorimetric method with detection wavelengths of 560 nm and 545 nm for amylose and amylopectin, respectively [24].

### 2.3. In Vitro Digestion Experiment

The in vitro digestion procedure was based on the method of van Kempen with minor adjustments [25]. Briefly, a 1.0 g feedstuff sample was weighed into a 50 mL conical flask. A solution containing 0.05 g of pepsin and 0.05 g of guar gum in 10 mL of HCl was added to each flask. The flasks were sealed with a membrane and incubated in a thermostatic water bath oscillator at 39 °C under shaking conditions at 200× *g* for 120 min. Subsequently, 10 mL of sodium acetate buffer (0.25 mol/L) and 5 mL of a mixed digestive enzyme mixture (containing 0.7 g of pancreatin, 0.728 mg of amyloglucosidase, and 1 mg of invertase) were added to each flask. The samples were further incubated in a thermostatic water bath oscillator at 39 °C for 480 min.

The digestion samples were collected at 0, 10, 20, 30, 60, 90, 120, 180, 240, 360, and 480 min of incubation. After centrifugation at 3500× *g* for 10 min, the supernatant was stored at −20 °C for glucose and total amino acid detection.

The starch digestibility, starch hydrolysis rate, and amino acid release rate were calculated as follows:

Starch digestibility (%) = (Gt × 0.9)/TS × 100%;

Starch hydrolysis rate (%/min) = (A_2_ − A_1_)/(t_2_ − t_1_);

Gt = glucose release at time t (mg);

0.9 = starch-to-glucose conversion factor;

TS = total starch content (mg);

A_1_, A_2_ = starch digestibility at times t_1_ and t_2,_ respectively;

Amino acid release rate (%) = AAt/AA480;

AAt = total amino acid release at time t (absolute quantity);

AA480 = total amino acid release at time 480 min (absolute quantity).

### 2.4. Determination of Total Amino Acid (TAA) and Glucose Contents

The total amino acid content was measured via the Total Amino Acid Assay Kit (A026-1-1; Nanjingjiancheng, Nanjing, China). The glucose content was measured via a glucose assay kit (E1010, Pulei, Beijing, China) according to the manufacturer’s instructions.

### 2.5. Determination of Serum Free Amino Acids

The concentrations of free amino acids were detected via ultra-performance liquid chromatography coupled with mass spectrometry (UPLC-MS). Serum samples were vortexed and centrifuged at 4 °C and 5000 rpm for 5 min. Two hundred microliters of the supernatant was transferred to a 1.5 mL centrifuge tube, followed by the addition of 8 μL of 2.5 mM ^2^H_8_-phenylalanine (as an internal standard) and 800 μL of methanol. The mixture was vortexed and centrifuged at 4 °C and 14,000 rpm for 10 min. A total of 500 μL of the supernatant was collected and dried under vacuum at 45 °C. The dried residue was redissolved in 100 μL of boric acid buffer. Next, 10 μL of the reconstituted sample was mixed with 50 μL of boric acid buffer and 20 μL of derivatization reagent. The mixture was vortexed immediately and incubated at room temperature for 1 min, and then sealed and heated in an oven at 55 °C for 10 min. After cooling to room temperature, the sample was filtered through a 0.22 μm filter. Chromatographic separation was performed using a Waters ACQUITY UPLC BEH C_18_ column (2.1 mm × 100 mm, 1.7 μm, Waters Corporation, Milford, MA, USA) with a guard column (2.1 mm × 5 mm, 1.7 μm, Waters Corporation, Milford, MA, USA). The column temperature was maintained at 35 °C, and the injection volume was 2 μL. The mobile phase was delivered at a flow rate of 0.3 mL/min via a gradient elution program, as shown in Table 1. The total run time was 15 min.

### 2.6. In Vivo Amino Acid Release Kinetics Experiment

#### 2.6.1. Animal Ethics Statement

The experiment was conducted at China Agricultural University in compliance with the Chinese guidelines for animal welfare and approved by the Institutional Animal Care and Use Committee of China Agricultural University (AW10305202-1-02).

#### 2.6.2. Experimental Design

Four healthy crossbred *Duroc* × *Landrace × Yorkshire* barrows with an average body weight of 28.05 kg were surgically fitted with cannulas in the portal vein, femoral artery, and femoral vein. Pigs were housed individually in metabolic cages (1.27 m × 0.90 m × 0.50 m, Chengdejiuyun Agricultural and Livestock Co., Ltd., Chengde, China) in a temperature-controlled room with free access to water throughout the experiment. The daily feed intake was determined based on 4% of the average body weight of the pigs. Pigs were allotted to a 4 × 4 Latin square design and fed semi-purified experimental diets with corn, soybean meal, wheat, or wheat bran as the sole nitrogen source. The experimental diets were formulated to meet the nutritional requirements of the pigs (Table 2).

During the sampling period, pigs were subjected to an overnight fast, and the experimental diet was fed at 08:30 the following morning. Blood samples were collected from the portal vein, femoral artery, and femoral vein cannula at 0, 0.5, 1.5, 2.5, 3.5, 4.5, 5.5, and 6.5 h postfeeding into EDTA-coated anticoagulant tubes. The samples were incubated at room temperature for 1 h, centrifuged at 3000 rpm for 10 min, and then stored at −20 °C for analysis of amino acid concentrations.

### 2.7. Statistical Analysis

Two-tailed Student’s tests were used for single comparisons and analysis of variance (ANOVA) with LSD tests for multiple comparisons. Statistical analysis was performed using SPSS Statistics 26 software, and graphics were constructed with GraphPad Prism 9.5 software.

## 3. Results

### 3.1. Nutritional Composition Analysis of Energy and Protein Feedstuffs

To comprehensively elucidate the nutritional profiles of energy and protein feedstuffs, nine commonly used energy feedstuffs and nineteen protein feedstuffs were collected. The gross energy, dry matter, crude protein, crude fat, crude fiber, neutral detergent fiber, and acid detergent fiber contents were analyzed (Table 3 and Table 4). In addition, total starch and the ratio of amylopectin to amylose were analyzed for the energy feedstuffs (Table 3 and Table 4). Among the nine energy feedstuffs, corn starch presented the highest total starch content, whereas wheat bran presented the lowest. The ratio of amylopectin to amylose varied significantly among the energy feedstuffs. Wheat bran also had the highest amylopectin/amylose ratio, and sorghum had the lowest amylopectin/amylose ratio, suggesting a greater proportion of amylose in its starch composition. Among the nineteen energy feedstuffs, whey protein powder had the lowest protein content at 3.81%, whereas hydrolyzed feather meal had the highest protein content at 86.47%. Notably, extruded soybeans presented high levels of both crude fat and crude fiber. These findings highlight the substantial differences in nutritional composition among feedstuffs, which may have implications for their nutritional value and digestibility in animal diets.

### 3.2. In Vitro Enzyme Hydrolysis Curve and Characteristics of Energy Feedstuffs

**Figure 1:**
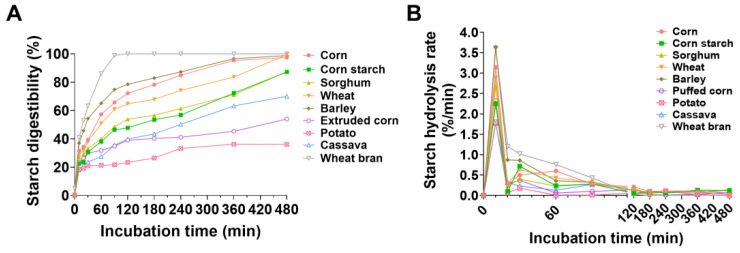
(**A**) Starch digestibility and (**B**) hydrolysis rate in energy feedstuffs.

The starch digestibility of all the feedstuffs increased progressively with prolonged incubation time. After incubation for 480 min, wheat bran presented the highest starch digestibility (100%), followed by wheat (99.42%), barley (98.83%), corn (97.40%), sorghum (87.44%), corn starch (87.24%), cassava (70.09%), extruded corn (54.04%), and potato (36.11%) (Figure 1A). The starch hydrolysis rate reached its peak at 10 min of incubation for all feedstuffs, with the highest rate observed in barley (3.64%), followed by wheat bran (3.61%), while the lowest rate was in cassava (1.77%) (Figure 1B). After 20 min of incubation, the starch hydrolysis rate decreased to varying extents, with the largest decrease observed in corn and the smallest in cassava (Figure 1B). After 30 min of incubation, starch hydrolysis rates continued to decrease in barley and cassava but showed slightly increases in the other feedstuffs. After 30 min, the starch hydrolysis rate gradually decreased with prolonged incubation time, and the cumulative release of total glucose increased slowly, with wheat showing the greatest increase and potato showing the lowest increase (Figure 1B).

### 3.3. Multiple Linear Regression Model of Starch Digestibility on the Basis of Nutrient Composition of Energy Feedstuffs

By analyzing the correlation between the in vitro starch digestibility and nutritional components of the feedstuffs, a multiple linear regression prediction model was developed. The results indicated that the content of crude fiber and total starch and the ratio of amylopectin to amylose were the primary predictors of in vitro starch digestibility. Crude fiber demonstrated a positive correlation with starch digestibility, with this enhancing effect becoming more pronounced as incubation time increased. In contrast, total starch showed an inverse relationship with starch digestibility, although this inhibitory effect diminished progressively with extended incubation. The ratio of amylopectin to amylose showed two distinct effects over incubation time: it increased starch digestibility in the first 4 h, but this effect slowly decreased and eventually became negative at 6 h and 8 h of incubation. The model parameters were presented in Table 5.

### 3.4. In Vitro Enzyme Hydrolysis and Amino Acid Release Characteristics of Protein Feedstuffs

**Figure 2:**
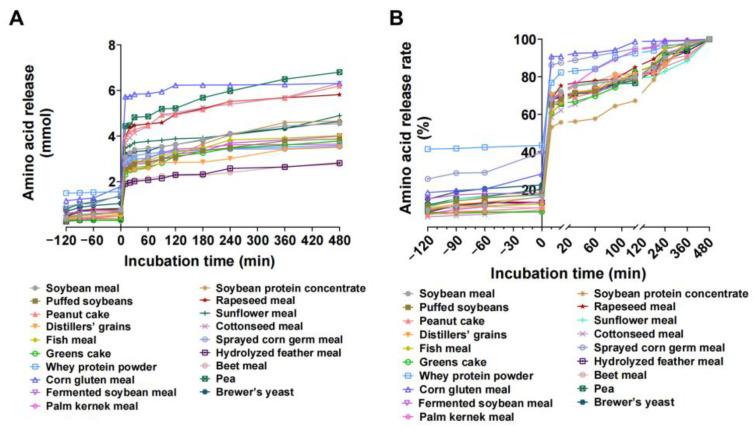
(**A**) Total amino acid accumulation release and (**B**) release rate in protein feedstuffs.

The release of total amino acids during in vitro digestion of 19 protein feedstuffs was significantly different. With the same weight of feedstuff (1.0 g), a total amino acid release of more than 6 mmol included pea (6.80 mmol, the highest), cottonseed meal, corn gluten meal, peanut cake, and corn gluten meal, whereas the hydrolyzed feather meal and beet pulp (2.76 mmol, the lowest) had total amino acid release values less than 3 mmol (Figure 2A).

During the gastric digestion phase, the release of total amino acids from various feedstuffs increased steadily at a relatively low rate. After 2 h of gastric digestion, corn gluten powder had the greatest amount of total amino acid release, whereas beet meal had the lowest amount (Figure 2B).

Upon entering the intestinal digestion phase, the rate of total amino acid release accelerated, peaked at 10 min and then decreased rapidly. At 10 min of intestinal incubation, corn gluten meal achieved the highest total amino acid release (90.81%), whereas soy protein concentrate had the lowest total amino acid release (53.13%) (Figure 2B). During the mid-intestinal incubation period (10–120 min), the cumulative release of total amino acids increased gradually. After 120 min of intestinal incubation, corn gluten meal had the highest total amino acid release (98.75%), followed by sprayed corn germ meal (95.00%) and palm kernel meal (94.14%). After 120–480 min of intestinal incubation, the greatest increase in total amino acid release was observed in the soy protein concentrate (32.77%), followed by the pea concentrate (23.32%) (Figure 2B). These results highlight the distinct amino acid release profiles of different protein feedstuffs during in vitro digestion.

### 3.5. In Vivo Amino Acid Metabolism and Deposition Characteristics

**Figure 3:**
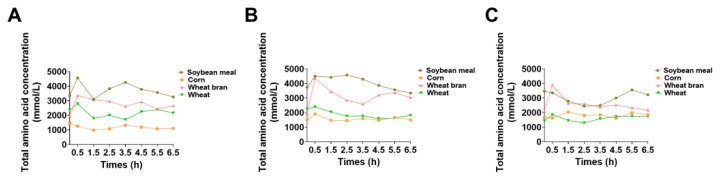
(**A**) Variation in the total amino acid concentration of pigs in the portal vein, (**B**) femoral artery, and (**C**) femoral vein after feeding.

**Figure 4:**
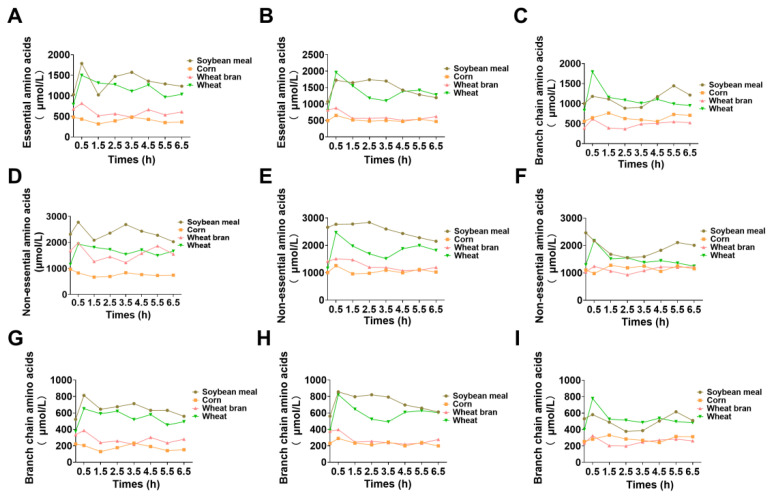
(**A**) Variation in the essential amino acid concentration of pigs in the portal vein, (**B**) femoral artery, and (**C**) femoral vein after feeding. (**D**) Variation in the non-essential amino acid concentration of pigs in the portal vein, (**E**) femoral artery, and (**F**) femoral vein after feeding. (**G**) Variation in branch chain amino acids concentration of pigs in the portal vein, (**H**) femoral artery, and (**I**) femoral vein after feeding.

To elucidate the amino acid metabolic and deposition characteristics of different feedstuffs in growing pigs, blood samples were collected from the portal vein, femoral artery, and femoral vein at various time points postfeeding. The concentrations of total amino acids and individual free amino acids in the blood were measured. In pigs fed a diet with soybean meal as the sole nitrogen source, the total amino acid concentration in the portal vein fluctuated within the first 2.5 h postfeeding and then gradually decreased thereafter (Figure 3). In contrast, the total amino acid concentration in the femoral artery exhibited a slow initial increase followed by a gradual decline. The concentration in the femoral vein showed the opposite trend, with a slow initial decrease followed by a gradual increase (Figure 3). For pigs fed a diet with corn as the sole nitrogen source, the total amino acid concentration in the portal vein peaked at 3.5 h postfeeding and then slowly declined to a stable level. The total amino acid concentrations in the femoral artery and femoral vein peaked at 0.5 and 1.5 h postfeeding, respectively (Figure 3). In pigs fed a diet with wheat bran as the sole nitrogen source, the total amino acid concentrations in the portal vein, femoral artery, and femoral vein all peaked at 0.5 h postfeeding. The concentration in the portal vein subsequently exhibited minor fluctuations, whereas the femoral artery concentration gradually decreased, and the femoral vein concentration initially tended to decrease but then slowly increased (Figure 3). In pigs fed a diet with wheat as the sole nitrogen source, the total amino acid concentrations in all three blood vessels peaked at 0.5 h postfeeding, similar to those in the wheat bran group. After this peak, the total amino acid concentration in the portal vein initially decreased and then fluctuated, whereas the concentrations in the femoral artery and femoral vein rapidly decreased and remained stable between 1.5 and 6.5 h postfeeding (Figure 3). In addition, significant differences were observed in the concentrations of essential amino acids, nonessential amino acids, and branched-chain amino acids (Figure 4).

### 3.6. Portal Vein Amino Acid Release Fitting Model Based on Chemical Components and In Vitro Digestion

Through analysis of the correlations among portal vein amino acid release, in vitro amino acid release, and nutritional components of the feedstuffs, a multiple linear regression prediction model was developed. The results indicated that the contents of DM, CP, NDF, EE, GE, total starch, and in vitro digestion were the primary predictors of portal vein amino acid release. In the model, GE had a large effect on portal vein amino acid release, followed by DM and EE, and the effect of in vitro amino acid release was relatively small. The model parameters were presented in Table 6.

## 4. Discussion

Starch and protein are two crucial nutritional components in feedstuffs that play vital roles in animal metabolism. Starch is hydrolyzed into glucose, which serves as the primary substrate for energy metabolism, while protein is broken down into amino acids, providing the fundamental building blocks for the synthesis of body tissues, enzymes, and hormones. The normal supply of glucose and amino acids is essential for maintaining normal physiological functions and ensuring efficient livestock production. Synchronized digestion and absorption of starch and protein are critical factors influencing the utilization efficiency of glucose and amino acids [26,27,28]. Therefore, this study investigated the release patterns of glucose and amino acids from different energy and protein sources through in vitro digestion and in vivo blood cannulation experiments.

Our findings demonstrated that feedstuffs with different compositions exhibit distinct patterns of glucose and amino acid release. In this study, all the feedstuffs demonstrated peak digestion rates at 10 min of intestinal incubation, followed by a rapid decline. After that, the rate of digestion gradually decreased, and eventually, the digestibility slowly increased. Subsequently, the digestion rate gradually decreased while digestibility showed a slow but steady increase. This pattern may be attributed to the mechanism of enzymes during different digestive phases. In the gastric digestion phase, pepsin primarily breaks peptide bonds of the large protein fraction. During the intestinal incubation, amyloglucosidase attacks starch granules, increasing pores and subsequently hydrolyzing them into glucose, while pancreatin further degrades long-chain peptides into small peptides and free amino acids [29,30]. As for energy feedstuffs, potato starch had the lowest digestibility, whereas wheat, corn, and barley starches had relatively high digestibility, exceeding 90%. These results are consistent with those of previous studies by Lee and Weurding [11,31], likely because of the characteristics of starch granules. Potato starch granules have diameters of 10–50 μm, which are larger than those of most cereals and cassava starches, whereas corn, barley, and wheat starch granules range from 2 to 20 μm in diameter [32,33]. Larger starch granules have smaller surface areas exposed to digestive enzymes, making them more resistant to enzymatic degradation. In addition to granule size, the digestibility of starch is also related to the proportion of amylopectin. Previous studies have shown that starch digestibility is associated with the amylose content and amylopectin structure [34,35]. In our study, the higher amylopectin-to-amylose ratio in barley, wheat, and corn than in sorghum, cassava, and potato contributed to the differences in digestibility. Non-starch components, particularly lipids and fibers, inhibit starch digestion by altering the gelatinization, molecular structure, enzymatic activity, and accessibility. In this study, the lower digestibility and digestion rates of extruded corn compared with the corn may result from the structure of the starch–lipid complex or the increased formation of resistant starch during storage [36,37,38]. Lipid and starch form complexes due to the formation of noncovalent interactions. They change rheological properties and limit carboxylic digestion. In cereal feeds, crude fiber primarily exists in the form of cellulose, which, along with the protein matrix, tightly encapsulates starch granules, restricting the combination of starch granules with water molecules and starch swelling [39]. The lower digestibility of sorghum observed in this study supports this notion.

Different protein feedstuffs exhibit distinct digestion patterns due to variations in amino acid composition, protein structure, and solubility. In this study, we evaluated the in vitro digestion of 19 protein sources and reported that, under the same weight conditions, peas released the greatest total amount of amino acids, whereas beet pulp released the least. This phenomenon may be attributed to the balanced and rich amino acid composition of peas, with proteins consisting mainly of soluble globulins [40]. In contrast, beet pulp has a relatively low protein content, resulting in a significant difference in the total amino acid released. Corn gluten meal rapidly released total amino acids, with the highest release rate in the first 10 min of small intestinal digestion, reaching 90.81% after 90 min. This result is consistent with Jennings’ trial [41]. Chen et al. [42] reported a decreasing order of digestibility for whey protein, soy protein isolate, and yeast concentrate. Similarly, in our study, the release rate of whey protein powder was greater than that of soy protein concentrate. The total amino acid release from the soy protein concentrate only began to increase gradually after 120 min of small intestinal digestion. This slower digestion rate may be due to protein aggregation during processing, which reduces protein solubility [43].

The total amino acid concentration in the portal vein of the corn group peaked at 3.5 h postfeeding (1318 μmol/L), whereas the concentrations in the wheat and bran groups peaked within 0.5 h (bran portal vein, 2797 μmol/L; wheat portal vein, 3369.66 μmol/L). These differences are likely closely related to the chemical composition and digestive characteristics of the feedstuffs. The large protein content of bran and wheat may account for the high amino acid content in the early stages of digestion [44]. In contrast, the higher starch content and dense endosperm structure of corn may delay the breakdown of the protein matrix, resulting in a delayed peak in amino acid release. The amino acid concentration in the soybean meal group fluctuated significantly within 2.5 h, which may be associated with its high crude protein content and antinutritional factors (e.g., trypsin inhibitors) that can temporarily inhibit digestive enzyme activity and prolong the protein digestion time. Regression models further indicated that in vivo amino acid release is significantly influenced by nutritional indicators such as DM, EE, and GE, as well as by in vitro release data. These findings suggest that fibers may inhibit the rapid release of amino acids in the early stages through a physical barrier effect, which is consistent with previous studies [16,45]. Additionally, crude fat had a significant positive effect on the 90 min model, likely because fat promoted bile secretion and emulsification, indirectly enhancing protein hydrolysis efficiency. The differences in amino acid concentration patterns among various blood vessels (portal vein, femoral artery, and femoral vein) suggest that, in addition to feed characteristics, blood circulation and metabolic regulation also influence amino acid distribution. For example, the stable concentration of amino acids in the femoral vein of the bran group after 1.5 h, despite fluctuations in the portal vein, may reflect the regulation of the liver on amino acid metabolism [46]. The rapid decline in the femoral artery concentration of the wheat group may be related to its high proportion of branched-chain amino acids, which are more readily taken up by peripheral tissues. The regression models developed in this study provide a practical tool for predicting in vivo amino acid release from feedstuffs. However, since the models were calibrated using specific energy and protein feedstuffs, the accuracy for other feed categories needs further testing. For example, processing methods such as extrusion can alter the fiber structure and digestibility, and anti-nutritional factors will prevent enzymes from working [16,17,18]. In addition, the effect of gut microbiota has not been taken into consideration in this study. The presence of resistant starch induces changes in the composition of microbiota and microbial metabolites. Microbial fermentation may alter the metabolic fate of nutrients that escape small intestinal digestion, thereby influencing systemic nutrient availability and nitrogen balance. Future studies should combine portal vein sampling with cecal content analysis and metagenomic profiling to quantify microbial contributions [47,48,49]. Thereby, future research could focus on calibrating model parameters for different processing methods and expanding the regression models to include microbial fermentation parameters to improve prediction accuracy and provide a theoretical basis for precise feed formulation.

## 5. Conclusions

In general, rapidly digestible feedstuffs (e.g., barley and corn gluten meal) can quickly release glucose and amino acids in the body, which is beneficial for improving amino acid absorption and utilization when sufficient glucose is available. In contrast, slowly digestible feedstuffs (e.g., potato) exhibit slower glucose release, which may lead to increased amino acid oxidation and reduced absorption efficiency. Therefore, understanding the digestion characteristics and nutrient release rates of feedstuffs is highly important for improving nitrogen use efficiency and growth performance in animals. This study provides data supporting the in vitro and in vivo release patterns of different feedstuffs, but has certain limitations. For example, starch and protein feedstuffs with different release rates were not matched to verify the interaction between glucose and amino acid release and utilization. Additionally, the role of the microbiota in intestinal nutrient metabolism cannot be overlooked. Further research is needed to elucidate how different release rates of glucose and amino acids affect animal metabolism in the presence of the gut microbiota.

## Figures and Tables

**Table 1:** Gradient elution procedure of UPLC-MS.

Time (min)	Flow Rate (mL/min)	Mobile Phase A (%)	Mobile Phase B (%)
0.0	0.3	97	3
3.0	0.3	93	7
3.8	0.3	88	12
8.0	0.3	66	34
8.9	0.3	30	70
12.0	0.3	97	3
15.0	0.3	97	3

**Table 2:** Composition of experimental diets.

Items	Diets (as Fed Basis)			
	Corn	Soybean Meal	Wheat	Wheat Bran
Corn (%)	96.70	0.00	0.00	0.00
Soybean meal (%)	0.00	33.00	0.00	0.00
Wheat (%)	0.00	0.00	96.70	0.00
Wheat bran (%)	0.00	0.00	0.00	30.00
Corn starch (%)	0.00	47.00	0.00	50.00
Soybean oil (%)	0.00	3.00	0.00	3.00
Sucrose (%)	0.00	15.00	0.00	15.00
Dicalcium phosphate (%)	1.50	0.90	1.50	0.90
Salt (%)	0.30	0.30	0.30	0.30
Limestone (%)	1.00	0.30	1.00	0.30
Premix (%)	0.50	0.50	0.50	0.50
Total (%)	100.00	100.00	100.00	100.00
Nutrient levels				
Net Energy (MJ/kg)	2676.66	3216.29	2483.26	3126.50
Dry matter (%)	87.79	89.99	89.97	89.93
Crude protein (%)	7.75	14.95	12.79	5.68
Lys	0.17	0.87	0.31	0.17
Met	0.16	0.19	0.18	0.06
Thr	0.19	0.52	0.34	0.10
Trp	0.04	0.15	0.13	0.06
Val	0.31	0.61	0.55	0.17
Ile	0.19	0.57	0.43	0.13
Leu	0.78	0.98	0.80	0.28
Arg	0.26	1.05	0.55	0.29
His	0.18	0.38	0.30	0.12
Phe	0.26	0.60	0.65	0.15
Ala	0.42	0.57	0.41	0.17
Asp	0.35	1.48	0.56	0.26
Cys	0.16	0.17	0.28	0.09
Glu	1.05	2.13	4.10	0.85
Gly	0.19	0.51	0.47	0.17
Pro	0.66	0.63	1.33	0.32
Ser	0.28	0.66	0.50	0.17
Tyr	0.16	0.50	0.39	0.13

**Table 3:** Nutrient levels of energy feedstuffs.

Items	GE (MJ/kg)	DM (%)	CP (%)	EE (%)	CF (%)	NDF (%)	ADF (%)	Total Starch (%)	Amylopectin/Amylose
Corn	16.01	88.11	8.23	3.62	1.81	6.56	1.62	71.64	1.71
Corn starch	14.48	86.38	1.05	ND	ND	0.14	ND	87.15	1.53
Sorghum	16.31	87.83	9.75	4.35	2.33	8.85	2.31	65.03	0.81
Wheat	16.06	88.31	15.31	1.99	1.84	10.78	1.79	62.58	1.77
Barley	16.63	90.36	12.19	2.23	3.80	20.10	6.26	53.14	2.31
Extruded corn	16.45	88.93	8.34	1.77	1.57	7.18	1.41	73.31	4.16
Potato	14.58	90.68	10.18	1.11	2.00	2.49	1.44	63.25	0.89
Wheat bran	16.55	89.03	17.15	2.67	10.73	38.37	10.66	18.35	5.82
Cassava	14.55	89.27	2.07	1.04	2.52	2.40	1.18	77.81	0.38
SEM	0.31	0.44	1.79	0.41	1.09	3.97	1.20	6.54	0.59

Notes: GE, gross energy; DM, dry matter; CP, crude protein; EE, ether extract; CF, crude fiber; NDF, neutral detergent fiber; ADF, acid detergent fiber; ND, not detected.

**Table 4:** Nutrient levels of protein feedstuffs.

Items	GE (MJ/kg)	DM (%)	CP (%)	EE (%)	CF (%)	NDF (%)	ADF (%)
Soybean meal	17.47	92.11	45.53	2.05	8.78	14.28	6.70
Extruded soybeans	21.48	93.55	36.57	16.49	17.91	21.55	10.45
Peanut cake	18.17	90.78	29.13	4.99	8.41	13.73	7.93
Distillers’ grain	18.66	87.79	27.94	7.42	8.92	24.38	7.20
Fish meal	17.94	91.84	68.73	5.52	1.67	18.96	2.53
Greens cake	18.36	91.08	37.15	7.56	13.55	23.20	16.18
Whey protein powder	14.00	95.27	3.81	2.48	ND	0.31	ND
Corn gluten meal	12.07	92.68	48.14	1.70	10.00	33.96	12.04
Fermented soybean meal	17.56	91.81	52.72	1.34	7.45	21.62	8.52
Palm kernel meal	17.49	90.51	17.89	5.47	16.63	57.71	32.00
Soybean protein concentrate	18.81	93.27	63.99	1.18	3.95	14.52	4.09
Rapeseed meal	17.97	89.38	41.62	3.47	10.13	25.43	13.68
Sunflower meal	17.29	90.70	41.08	1.16	12.65	23.25	14.25
Cottonseed meal	17.11	88.87	58.14	1.74	4.77	12.90	5.52
Sprayed corn germ meal	17.28	92.62	29.77	1.77	7.36	30.15	7.46
Hydrolyzed feather meal	22.22	92.38	86.47	4.60	1.32	29.21	4.90
Beet meal	14.99	90.71	8.65	0.57	15.47	34.23	19.76
Pea	17.69	93.43	31.66	3.99	6.36	24.11	12.22
Brewer’s yeast	17.45	92.26	43.69	0.55	ND	8.98	3.24
SEM	0.52	0.41	4.65	0.86	1.19	2.77	1.68

Notes: ND, not detected.

**Table 5:** Multiple linear regression modeling of starch digestibility based on nutrient composition of energy feedstuffs.

Items	Modeling	R^2^
0.5 h	SD0.5 = 3.969 × CF − 0.751 × TS + 3.242 × AA + 70.290	0.96
1 h	SD1.0 = 4.961 × CF − 0.632 × TS + 2.769 × AA + 67.815	0.87
2 h	SD2.0 = 6.889 × CF − 0.301 × TS + 1.922 × AA + 53.145	0.77
3 h	SD3.0 = 7.569 × CF − 0.205 × TS + 1.386 × AA + 49.319	0.74
4 h	SD4.0 = 9.084 × CF − 0.106 × TS + 0.117 × AA + 46.392	0.74
6 h	SD6.0 = 10.716 × CF − 0.175 × TS − 1.306 × AA + 34.560	0.67
8 h	SD8.0 = 11.425 × CF − 0.354 × TS − 1.308 × AA + 27.850	0.61

Notes: SD, starch digestibility; CF, crude fiber; TS, total starch; AA, amylopectin/amylose.

**Table 6:** Portal vein amino acid release fitting model based on chemical components and in vitro digestion.

Items	Modeling	R^2^
0.5 h	PR30min = 429.143 × DM − 10.833 × CP − 46.567 × NDF + 0.089 × R0 − 37,965.420	0.95
1.5 h	PR90min = 912.650 × DM + 1608.709 × EE − 129.991 × NDF + 0.067 × R1.5 − 88,252.98	0.99
2.5 h	PR150min = 7035.103 × GE − 282.173 × EE + 107.339 × TS + 0.028 × R2.0 − 119,520.792	0.99
3.5 h	PR210min = 511.211 × DM + 3979.605 × GE + 57.622 × TS + 0.004 × R3.0 − 111,495.633	0.93
4.5 h	PR270min = 4813.429 × GE + 73.348 × TS − 583.392 × EE + 0.022 × R4.0 − 80,244.804	0.99
5.5 h	PR330min = 2979.714 × GE − 1045.729 × DM + 24.195 × CP + 0.007 × R6.0 + 45,125.873	0.98
6.5 h	PR390min = 58.115 × DM − 945.941 × GE − 93.759 × TS − 0.059 × R8.0 + 25,526.378	0.99

Notes: PR, portal vein amino acid release; DM, dry matter; CP, crude protein; NDF, neutral detergent fiber; R, in vitro amino acid release; EE, ether extract; GE, gross energy; TS, total starch.

## Data Availability

The original contributions presented in this study are included in the article. Further inquiries can be directed to the corresponding authors.

## References

[1] Knudsen K.E.B., Lærke H.N., Ingerslev A.K., Hedemann M.S., Nielsen T.S., Theil P.K. (2016). Carbohydrates in pig nutrition-recent advances. J. Anim. Sci..

[2] Yang Y., Cai S., Huang F., Mo C., Wu Y., Cao J., Chen S., Wen Z., Liao X. (2025). Antibiotic resistance profile of nitrogenmetabolizing microbes in anoxic-oxic processes for swine wastewater treatment. Npj Clean Water.

[3] Yang Y., Cai S., Mo C., Dong J., Chen S., Wen Z. (2025). Profiles of antibiotic resistome risk in diverse water environments. Commun. Earth Environ..

[4] Martens B.M.J., Flecher T., de Vries S., Schols H.A., Bruininx E.M.A.M., Gerrits W.J.J. (2019). Starch digestion kinetics and mechanisms of hydrolysing enzymes in growing pigs fed processed and native cereal-based diets. Br. J. Nutr..

[5] Nadia J., Bronlund J., Singh R.P., Singh H., Bornhorst G.M. (2021). Structural breakdown of starch-based foods during gastric digestion and its link to glycemic response: In vivo and in vitro considerations. Comp. Rev. Food Sci. Food Safe.

[6] Li J., Tan B., Tang Y., Liao P., Yao K., Ji P., Yin Y. (2018). Extraction and identification of the chyme proteins in the digestive tract of growing pigs. Sci. China Life Sci..

[7] Wenderlein J., Kienzle E., Straubinger R.K., Schöl H., Ulrich S., Böswald L.F. (2022). Morphology of starch particles along the passage through the gastrointestinal tract in laboratory mice fed extruded and pelleted diets. Animals.

[8] Ai Y., Hasjim J., Jane J. (2013). Effects of lipids on enzymatic hydrolysis and physical properties of starch. Carbohydr. Polym..

[9] Wang J., Sun Z. (2018). Study on the release kinetics of amino acids of different protein sourcesin growing pigs diets. J. Anim. Sci..

[10] Yuan L., Tang Y., Liu X. (2015). Research on the factors affecting digestibility of protein. Food Sci. Technol. Econ..

[11] Lee T., Huang Y., Chiang C., Chung T., Chiou P.W., Yu B. (2011). Starch characteristics and their influences on in vitro and pig prececal starch digestion. J. Agric. Food Chem..

[12] Deng J., Wu X., Bin S., Li T.J., Huang R., Liu Z., Liu Y., Ruan Z., Deng Z., Hou Y. (2010). Dietary amylose and amylopectin ratio and resistant starch content affects plasma glucose, lactic acid, hormone levels and protein synthesis in splanchnic tissues. J. Anim. Physiol. Anim. Nutr..

[13] Lu Z., Donner E., Yada R.Y., Liu Q. (2016). Physicochemical properties and in vitro starch digestibility of potato starch/protein blends. Carbohydr. Polym..

[14] Damiran D., Yu P. (2011). Molecular basis of structural makeup of hulless barley in relation to rumen degradation kinetics and intestinal availability in dairy cattle: A novel approach. J. Dairy Sci..

[15] Damiran D., Yu P. (2010). Structural makeup, biopolymer conformation, and biodegradation characteristics of a newly developed super genotype of oats (CDC SO-I versus conventional varieties): A novel approach. J. Agric. Food Chem..

[16] Manikpuri S., Kheto A., Sehrawat R., Gul K., Routray W., Kumar L. (2024). Microwave irradiation of guar seed flour: Effect on anti-nutritional factors, phytochemicals, in vitro protein digestibility, thermo-pasting, structural, and functional attributes. J. Food Sci..

[17] Cao H., Huang Q., Wang C., Guan X., Huang K., Zhang Y. (2023). Effect of compositional interaction on in vitro digestion of starch during the milling process of quinoa. Food Chem..

[18] Axentii M., Codină G.G. (2024). Exploring the nutritional potential and functionality of hemp and rapeseed proteins: A review on unveiling anti-nutritional factors, bioactive compounds, and functional attributes. Plants..

[19] Berrocoso J.D., García-Ruiz A., Page G., Jaworski N.W. (2020). The effect of added oat hulls or sugar beet pulp to diets containing rapidly or slowly digestible protein sources on broiler growth performance from 0 to 36 days of age. Poult Sci..

[20] Zhou J., Tu J., Wang L., Yang L., Yang G., Zhao S., Zeng X., Qiao S. (2022). Free amino acid enriched diets containing rapidly but not slowly digested carbohydrate promote amino acid absorption from intestine and net fluxes across skeletal muscle of pigs. J. Nutr..

[21] Yin F., Zhang Z., Huang J., Yin Y. (2010). Digestion rate of dietary starch affects systemic circulation of amino acids in weaned pigs. Br. J. Nutr..

[22] Sun M., Zhao J., Wang X., Jiao H., Lin H. (2020). Use of encapsulated L-lysine-HCl and DL-methionine improves postprandial amino acid balance in laying hens. J. Anim. Sci..

[23] Luo C., Yu Y., Meng G., Yuan J. (2025). Slowly digestible starch impairs growth performance of broiler chickens offered low-protein diet supplemental higher amino acid densities by inhibiting the utilization of intestinal amino acid. J Anim Sci Biotechnol..

[24] He J., Yan F., Huang F., Xiao Y., Xie L. (2022). Determination of amylose and amylopectin contents in yam and taros by dual-wavelength spectrophotometry. Sci. Technol. Food Ind..

[25] van Kempen T.A.T.G., Regmi P.R., Matte J.J., Zijlstra R.T. (2010). In vitro starch digestion kinetics, corrected for estimated gastric emptying, predict portal glucose appearance in pigs. J. Nutr..

[26] Zhou J., Wang L., Yang L., Yang G., Zeng X., Qiao S. (2022). Different dietary starch patterns in low-protein diets: Effect on nitrogen efficiency, nutrient metabolism, and intestinal flora in growing pigs. J. Anim. Sci. Biotechnol..

[27] Yin D., Selle P.H., Moss A.F., Wang Y., Dong X., Xiao Z., Guo Y., Yuan J. (2019). Influence of starch sources and dietary protein levels on intestinal functionality and intestinal mucosal amino acids catabolism in broiler chickens. J. Anim. Sci. Biotechnol..

[28] Drew M.D., Schafer T.C., Zijlstra R.T. (2012). Glycemic index of starch affects nitrogen retention in grower pigs. J. Anim. Sci..

[29] Gong X., Hui X., Wu G., Morton J.D., Brennan M.A., Brennan C.S. (2022). In vitro digestion characteristics of cereal protein concentrates as assessed using a pepsin-pancreatin digestion model. Food Res. Int..

[30] Yang Z., Xu X., Singh R., de Campo L., Gilbert E.P., Wu Z., Hemar Y. (2019). Effect of amyloglucosidase hydrolysis on the multi-scale supramolecular structure of corn starch. Carbohyd. Polym..

[31] Weurding R.E., Veldman A., Veen W.A.G., Van Der Aar P.J., Verstegen M.W.A. (2001). Starch digestion rate in the small intestine of broiler chickens differs among feedstuffs. J. Nutr..

[32] Lynn A., Cochrane M.P. (1997). An evaluation of confocal microscopy for the study of starch granule enzymic digestion. Starch Stärke.

[33] Wang Y., Saulnier L., Ral J.-P., Falourd X., Kansou K. (2023). Determining whether granule structural or surface features govern the wheat starch digestion, a kinetic analysis. Carbohydr. Polym..

[34] Ramadoss B.R., Gangola M.P., Agasimani S., Jaiswal S., Venkatesan T., Sundaram G.R., Chibbar R.N. (2019). Starch granule size and amylopectin chain length influence starch in vitro enzymatic digestibility in selected rice mutants with similar amylose concentration. J. Food Sci. Technol..

[35] Khatun M.A., Razzak M., Hossain M.A., Rahman M.A., Khan R.A., Huque R. (2020). Gamma radiation application to rice: Reduced glycemic index in relation to modified carbohydrate observed in FTIR spectra. Curr. Res. Food Sci..

[36] Ye Y., Du H., Liu X., Jia G., Lu Z., Wang Y. (2025). Effects of different exogenous proteins combined with dry heat treatment on physicochemical properties and digestive characteristics of puffed corn flour. J. Sci. Food Agric..

[37] Liu Q., Liu Q., Yang Y., Jiao A., Jin Z. (2024). Isothermal retrogradation preparation of type iii resistant starch from extruded-debranched starch: Structure and in vitro digestibility. Int. J. Biol. Macromol..

[38] Li Y., Zhang A.R., Luo H.F., Wei H., Zhou Z., Peng J., Ru Y.J. (2015). In vitro and in vivo digestibility of corn starch for weaned pigs: Effects of amylose:amylopectin ratio, extrusion, storage duration, and enzyme supplementation. J. Anim. Sci..

[39] Chen X., Zhu L., Zhang H., Wu G., Cheng L., Zhang Y. (2024). A review of endogenous non-starch components in cereal matrix: Spatial distribution and mechanisms for inhibiting starch digestion. Crit. Rev. Food Sci. Nutr..

[40] Lu Z.X., He J.F., Zhang Y.C., Bing D.J. (2020). Composition, physicochemical properties of pea protein and its application in functional foods. Crit. Rev. Food Sci. Nutr..

[41] Jennings J.S., Meyer B.E., Guiroy P.J., Cole N.A. (2018). Energy costs of feeding excess protein from corn-based by-products to finishing cattle. J. Anim. Sci..

[42] Chen Z., Zhang H., Zhang S., Zhang Y. (2019). Amino acid composition analysis and in vitro dynamic digestion of proteins from three different sources. J. Henan Univ. Technol. Nat. Sci. Ed..

[43] Zhao F., Guo X., Zhao S., Zhang M., Yang Y. (2023). Effects of processing conditions on the solubility and structure of alcoholeached soybean protein concentrate. China Oils Fats.

[44] Li Y., Wang L., Wang H., Li Z., Qiu J., Wang L. (2022). Correlation of microstructure, pore characteristics and hydration properties of wheat bran modified by airflow impact mill. Innov. Food Sci. Emerg..

[45] Heyer C.M.E., Wang L.F., Beltranena E., Zijlstra R.T. (2021). Nutrient digestibility of extruded canola meal in ileal-cannulated growing pigs and effects of its feeding on diet nutrient digestibility and growth performance in weaned pigs. J. Anim. Sci..

[46] Paulusma C.C., Lamers W.H., Broer S., van de Graaf S.F.J. (2022). Amino acid metabolism, transport and signalling in the liver revisited. Biochem. Pharmacol..

[47] Cronin P., Joyce S.A., O’Toole P.W., O’Connor E.M. (2021). Dietary fibre modulates the gut microbiota. Nutrients.

[48] Yang X., Darko K.O., Huang Y., He C., Yang H., He S., Li J., Li J., Hocher B., Yin Y. (2017). Resistant starch regulates gut microbiota: Structure, biochemistry and cell signalling. Cell Physiol. Biochem..

[49] Li H., Zhang L., Li J., Wu Q., Qian L., He J., Ni Y., Kovatcheva-Datchary P., Yuan R., Liu S. (2024). Resistant starch intake facilitates weight loss in humans by reshaping the gut microbiota. Nat. Metab..

